# Gm-PLoc: A Subcellular Localization Model of Multi-Label Protein Based on GAN and DeepFM

**DOI:** 10.3389/fgene.2022.912614

**Published:** 2022-06-15

**Authors:** Liwen Wu, Song Gao, Shaowen Yao, Feng Wu, Jie Li, Yunyun Dong, Yunqi Zhang

**Affiliations:** ^1^ Engineering Research Center of Cyberspace, Yunnan University, Kunming, China; ^2^ School of Software, Yunnan University, Kunming, China; ^3^ Yunnan Key Laboratory of Statistical Modeling and Data Analysis, School of Mathematics and Statistics, Yunnan University, Kunming, China

**Keywords:** protein subcellular localization, class imbalance learning, multi-label classification, generative adversarial networks, deep learning

## Abstract

Identifying the subcellular localization of a given protein is an essential part of biological and medical research, since the protein must be localized in the correct organelle to ensure physiological function. Conventional biological experiments for protein subcellular localization have some limitations, such as high cost and low efficiency, thus massive computational methods are proposed to solve these problems. However, some of these methods need to be improved further for protein subcellular localization with class imbalance problem. We propose a new model, generating minority samples for protein subcellular localization (Gm-PLoc), to predict the subcellular localization of multi-label proteins. This model includes three steps: using the position specific scoring matrix to extract distinguishable features of proteins; synthesizing samples of the minority category to balance the distribution of categories based on the revised generative adversarial networks; training a classifier with the rebalanced dataset to predict the subcellular localization of multi-label proteins. One benchmark dataset is selected to evaluate the performance of the presented model, and the experimental results demonstrate that Gm-PLoc performs well for the multi-label protein subcellular localization.

## 1 Introduction

Proteins are the crucial material basis of life activities, which participate in various biological activites (Qu et al., 2019). Previous researches show that almost all life phenomena are closely related to the structure and function of proteins, and the correct subcellular localization of proteins can assist biologists in understanding proteins (Wei et al., 2018; Zhang et al., 2021). Simultaneously, the subcellular localization of proteins also plays an essential role in disease diagnosis, drug design, and other biological researches (Li et al., 2020; Wang et al., 2021). Biological experiments (Murphy et al., 2000) were widely used to annotate protein subcellular localizations in the early stage of research. However, the cost of such conventional experiments is expensive (Yu B. et al., 2017; Liu et al., 2021). Therefore, the machine learning methods are introduced to solve the problems mentioned above, and have good results in protein subcellular localization (Wan et al., 2017), genomic island detection (Dai et al., 2018; Kong et al., 2020; Onesime et al., 2021) and so on. The machine learning based protein subcellular localization methods aim to learn the mapping relationship between protein and subcellular localization, so as to accurately predict the subcellular location of a given protein. According to the different forms of protein data, these methods can be divided into two categories: the sequence-based and the image-based.

1) The core idea of the sequence-based methods is to extract the feature information of each protein and build the classifier. One protein sequence is generally composed of 20 amino acids, and it is particularly important to extract the feature information of protein sequences. Therefore, some correlation methods concerning feature extraction have been proposed. Nakashima et al. introduced the amino acid composition (AAC) to represent features of proteins (Nakashima and Nishikawa, 1994), and each protein was digitized into a 20-dimensional vector, where each value represents the percentage of amino acids. AAC can express the global feature of amino acids, but it ignores the sequence of amino acids and the interaction between residues. In order to solve this problem, Petrilli et al. proposed the dipeptide composition (Petrilli, 1993), which comprehensively considered the relationship between amino acids. However, the physicochemical characteristics of amino acids have been ignored in the above methods. Therefore, Chou et al. proposed the pseudo-amino acid composition (PseAAC) (Chou, 2001) included the physicochemical characteristics and the order information of amino acids, which has been applied in predicting various protein attributes, such as protein subchloroplast locations (Sun and Du, 2021), protein sub-Golgi locations (Zhao et al., 2019) and so on. With further research, researchers leveraged the position specific score matrix (PSSM) to extract the evolutionary information of proteins. PSSM is the most commonly used method for the single feature representation. For the purpose of obtaining more rich information about protein sequences, the above mentioned methods of single feature representation have been fused to get the integrated information of proteins (Zakeri et al., 2011; Li et al., 2019; Liu et al., 2019; Ding et al., 2020).
2) The image-based methods construct the classification model with biological images, and the biological images mentioned here include Immunofluorescence (IF) and Immunohistochemistry (IHC) (Hu et al., 2021). There are some non-informative regions in the biological images, such as stroma, debris and background, the channel separation of DNA and protein is usually used to remove thus redundant information (Xu et al., 2019). With the continuous deepening of relevant researches, the neural network is introduced to extract the feature information of biological images. Long et al. proposed a new image-based method ImPloc for protein subcellular localization in 2020, and ImPloc used the convolutional neural network and transfer learning to extract feature information (Long et al., 2019). In the same year, Su et al. extracted feature information of biological images through five widely used neural network models, and achieved good research results (Su et al., 2020).

No matter what the form of protein data is, the ultimate aim is to accurately predict the subcellular localization based on relevant protein data. Research shows that a protein is usually located in one or more subcellular localizations (Xu et al., 2013; Cheng et al., 2019; Zhang et al., 2021; Shen et al., 2020), thus, the subcellular localization of these proteins can be abstracted to a multi-label classification. There are some classification models have been introduced or proposed, such as support vector machine (SVM) (Xu et al., 2009; Zhao et al., 2019; Yang et al., 2020; Sun and Du, 2021), deep neural networks (Semwal and Varadwaj, 2020; Su et al., 2020; Wang et al., 2021) and so on. The above methods are dedicated to improving the prediction accuracy of the subcellular localization from the aspects of feature representation and model construction, but some of these models have some limitations when dealing with the class imbalance problem. Due to the characteristics of protein data and environmental influences, the distribution of proteins annotated with subcellular localization is generally imbalanced, which means the number of proteins belonging to one subcellular localization is far less than that of others. The class imbalance problem of protein data seriously affects the model performance, especially when proteins have multiple subcellular localizations, and the conventional methods based on oversampling are difficult to handle the multiclass imbalanced problems effectively, such as the synthetic minority oversampling technique (SMOTE) (Buda et al., 2018). These methods alleviate data imbalance by inserting new samples between minority classes based on the sample spacing, which will further aggravate the problems of sample stacking and poor diversity.

For the mentioned problems, we propose a new model called Gm-PLoc to predict the subcellular localization of multi-label proteins in this paper. Firstly, PSSM is used to extract the evolutionary features (Gong et al., 2021). Secondly, a new method called generative adversarial networks for synthesizing minority samples (SM-GAN) is put forward based on generative adversarial networks (GAN), and then the PSSM of each protein will be fed into SM-GAN for rebalancing the dataset. Finally, a classification model called multi-label deep factorization machine (ML-DeepFM) is proposed to predict the subcellular localization of proteins based on deep factorization machine (DeepFM). For evaluating the performance of Gm-PLoc, we perform 10 fold cross validation on the benchmark dataset, and experimental results show that Gm-PLoc can effectively predict the subcellular localization of multi-label proteins.

The following content is arranged as follows. In Section 2, the proposed model Gm-PLoc will be introduced in detail. Section 3 provides a detailed analysis of the experimental results of Gm-PLoc on a benchmark dataset. The last is a summary of this paper and prospects for our future work.

## 2 Materials and Methodology

### 2.1 Benchmark Dataset

In this work, a benchmark dataset of human proteins (Shen and Chou, 2009) is utilized to evaluate the Gm-PLoc, which is collected from the Swiss-Port database with strict screening. This dataset contains 3,106 distinct samples from 14 classes, and the similarity between protein samples is less than 25% in each class. In the dataset, 2,580 samples belong to one location, 480 samples belong to two locations, 43 samples belong to three locations, and remaining 3 samples belong to 4 locations. Meanwhile, there is a vast difference in the sample size of each location. More dataset details can be found at http://www.csbio.sjtu.edu.cn/bioinf/hum-multi-2/Data.htm.

### 2.2 Overview of Gm-PLoc

**FIGURE 1:**
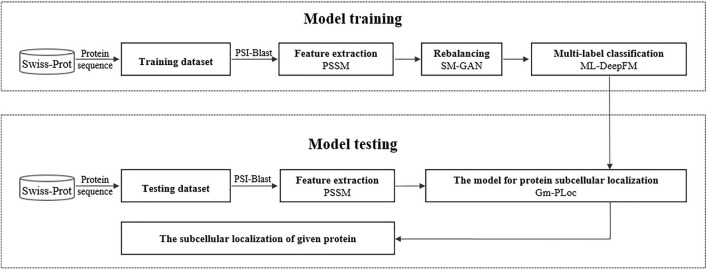
Predicting subcellular localization of a given protein by Gm-PLoc.

Gm-PLoc is proposed to predict the subcellular localization of multi-label proteins, which can handle the multiclass imbalanced problem. The construction of this model includes two processes: model training and model testing, as shown in Figure 1.

During the model training process, we use PSSM to extract features from protein sequences, then utilize a specially designed GAN called SM-GAN to synthesize pseudo samples for minority classes, and finally construct a multi-label classifier called ML-DeepFM with improved DeepFM for the protein subcellular localization. In the model testing, a given protein is fed into Gm-PLoc, and its relevant subcellular localizations will be output. In the next part, we will introduce the related knowledge for the procedure above in more detail.

#### 2.2.1 Proteins Sample Formulation

In the process of biological evolution, the similarity between proteins will fade away, but they still have some common properties. The evolutionary information of a protein sequence has a close relationship with protein physiological functions, and the PSSM is designed to capture the evolutionary information from protein sequences (Gong et al., 2021). The PSSM can be obtained by using position specific iterative-basic local alignment search tool (PSI-BLAST) to search similar proteins of a query protein in a non-redundant database (Murphy et al., 2000), and the parameters of E-value and iterations are set as 0.001 and 3, respectively.

For a query protein P including L number of residues, the corresponding PSSM can be denoted by a L×20 matrix as follows:

$$P_{pssm}=\left[\begin{array}{ccccc} E_{1\to 1} & \cdots & E_{1\to j} & \cdots & E_{1\to 20}\\ \vdots & \cdots & \vdots & \cdots & \vdots \\ E_{i\to 1} & \cdots & E_{i\to j} & \cdots & E_{i\to 20}\\ \vdots & \cdots & \vdots & \cdots & \vdots \\ E_{L\to 1} & \cdots & E_{L\to J} & \cdots & E_{L\to 20} \end{array}\right]$$ (1)

where $E_{i\to j}$ means the score of amino acid residue in the ith position being turned into amino acid of the jth type during the evolution process, and 20 stands for the number of native amino acid types.

The PSSM matrix resulting from Eq. 1 is not uniform with different length of protein sequences, which leads to the fact that matrix cannot be fed to the general machine learning model in a proper form. For this problem, the matrix dimensions are fixed to 20×20 by using a discrete method (Chou and Shen, 2008), and the PSSM matrix is formulated as follows:

$$\bar{P_{pssm}}={\left[\begin{array}{cccc} \bar{E_{1}} & \bar{E_{2}} & \cdots & \bar{E_{20}} \end{array}\right]}^{T}$$ (2)

where

$$\bar{E_{j}}=\frac{1}{L}\sum_{i=1}^{L}E_{i\to j}\left(j=1,2,\cdots ,20\right)$$ (3)

In Eq. 3, $\bar{E_{j}}$ denotes the average score of the amino acid residues in the jth protein P .

#### 2.2.2 Generative Adversarial Networks for Synthesizing Minority Samples (SM-GAN)

Most of the benchmark datasets utilized for training classifiers have the class imbalance problem in the protein subcellular localization, which seriously affects the performance of classifier. Different approaches are proposed and utilized to handle the class imbalance problem in previous researches (He and Garcia, 2009; Yin et al., 2020), among which oversampling methods are widely used, as they are more elastic and better in aiming at a specific problem. However, oversampling methods, such as SMOTE, are faced with the problems of sample stacking and poor diversity.

**FIGURE 2:**
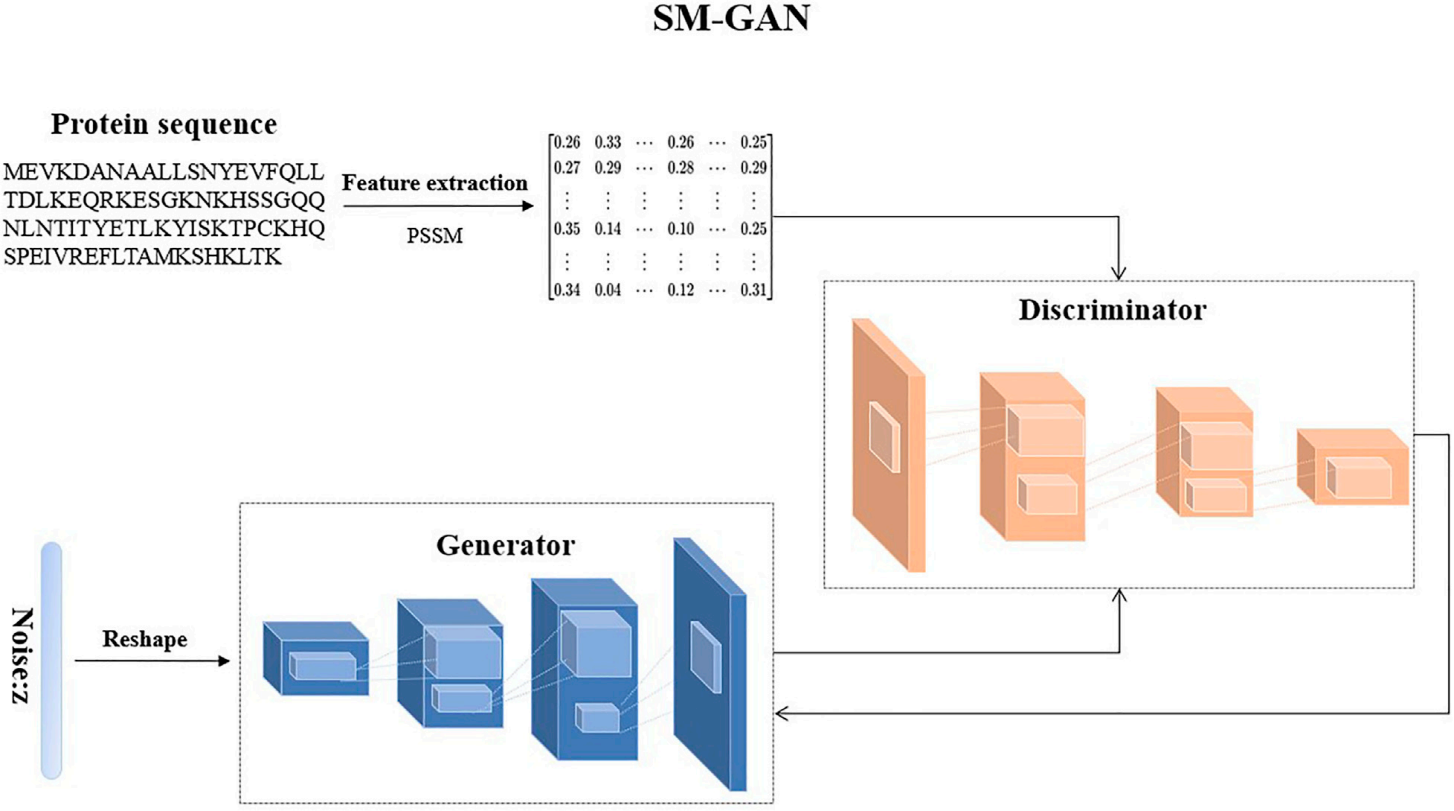
An illustration of SM-GAN.

To solve these problems, we design a special GAN called SM-GAN to generate pseudo samples of minority classes, and the illustration of SM-GAN is shown in Figure 2. It includes two models: a generative model G that learns the distribution of minority data, and a discriminative model D that measures the probability of a given sample coming from G . SM-GAN, in essence, is a zero sum game between G and D , which can generate samples represented by PSSM for minority classes. Note that the network structure of G and D are convolutional layers rather than fully connected layers, because each column in the PSSM matrix can be regarded as a time series feature, and the conventional fully connected layers will lose the potential spatial information in the PSSM matrix. This section will discuss how to use SM-GAN to deal with the class imbalance problem.

The purpose of D is to distinguish the real samples from the fake samples generated by G . At the same time, the purpose of G is to generate samples that confuse the discriminator D . Following this adversarial process, the generator G is expected to generate high-quality samples imitating the samples of minority classes. The generated samples should be as similar as possible to the real samples, but at the same time, these samples cannot be stacked together.

In the process of generating new samples by SM-GAN, the generator G randomly samples a noise vector z from latent space $P_{z}$ and produces the new sample $G_{z}$ ; then synthesized samples and real samples will be fed into the discriminator D , and then we train D to correctly distinguish fake sample $G_{z}$ from the real sample x . The object function of the discriminator can be presented as follows:

$$\underset{D}{\max}v_{\left(D\right)}=E_{x\in P_{data}\left(x\right)}\left[\log D_{x}\right]+E_{z\in P_{z}\left(z\right)}\left[\log\left(1-D\left(G\left(z\right)\right)\right)\right]+E_{z\in P_{z},x\in P_{data}\left(x\right)}\left[\log\left(d\left(G\left(z\right)-\bar{x}\right)\right)\right]$$ (4)

where $P_{data}$ means the distribution of real minority samples, D(x) denotes the probability of x being real, $\bar{x}$ represents the sample mean, $d\left(x_{f},\bar{x_{r}}\right)$ is a metric used to evaluate the distance between the synthesized samples and real samples. By maxing Eq. 4, D maximizes the distance between the generated sample and the real sample to further accurately identify the real sample. Now, we obtain the objective function of the discriminator as shown in Eq. 4, and then we train G to confuse D . The objective function of the generator is:

$$\underset{G}{\min}V\left(G,D\right)=E_{z\in P_{z}\left(z\right)}\left[-\log\left(D\left(G\left(z\right)\right)\right)\right]+\lambda E_{z\in P_{z},x\in P_{data}\left(x\right)}\left[\log\left(d\left(G\left(z\right)-\bar{x}\right)\right)\right]$$ (5)

where λ is a hyper parameter for the regulation of the distance between generated samples and real samples. Following Eqss 4, 5, we reach our objective of generating high-quality samples of the minority classes in protein subcellular localization.

#### 2.2.3 Multi-Label DeepFM (ML-DeepFM)

**FIGURE 3:**
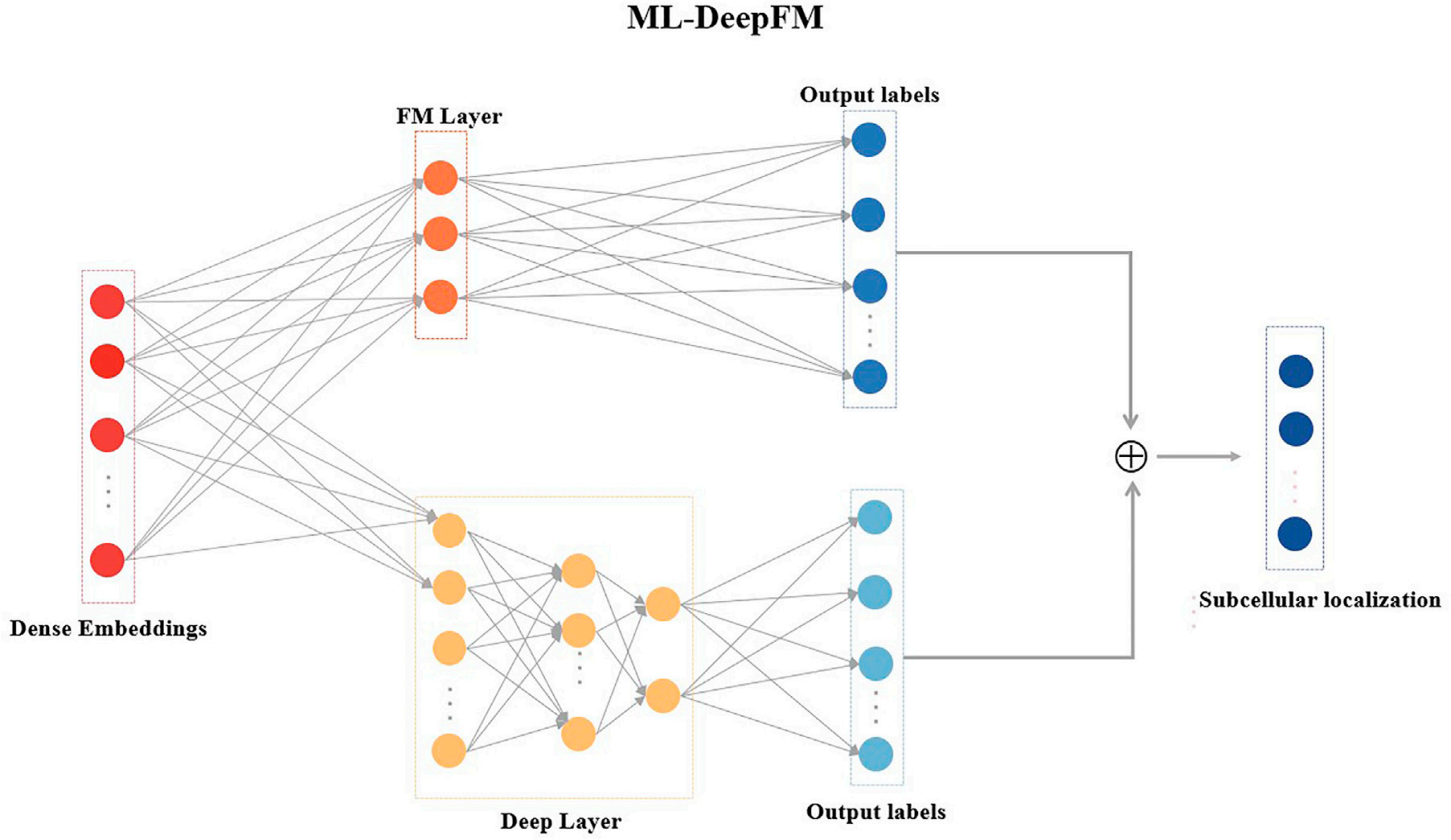
The model architecture of ML-DeepFM.

Multi-label classification is a common problem where a given sample has more than one label (Zhang and Zhou, 2014), which has attracted a lot of research attention. Many approaches are proposed for multi-label learning tasks, but the classification performance still needs to be improved. In this work, we design a multi-label classifier ML-DeepFM based on DeepFM (Guo et al., 2017) for the subcellular localization, and its structure is shown in Figure 3.

DeepFM has been extensively used in both the academic and industry because of its ability to learn low-order and high-order feature interactions. However, DeepFM cannot solve the problem of the multi-label classification for the protein subcellular localization. Therefore, ML-DeepFM is proposed, and its details will be described in this section.

ML-DeepFM includes two components: the FM component that models low-order feature interactions and the Deep component that extracts high-order feature interactions, as shown in Figure 3.

The FM component and Deep component must be trained in parallel, and then the predicted results of a given sample will be:

$$\hat{y}=y_{FM}+y_{Deep}$$ (6)

where $\hat{y}$ denotes the predicted labels, $y_{FM}$ and $y_{Deep}$ represent the output vectors of FM component and Deep component, respectively.

As shown in Eq. 6, for calculating the predicted label $\hat{y}$ , we need to get the output vectors of FM component and Deep component severally.

1) FM component: FM component is a factorization machine proposed by Steffen Rendle (Rendle, 2010), and it can capture the information of feature interactions effectively. Assuming that sample x includes n features, and each feature can be expressed as $x_{i},i\in n$ . For the feature $x_{i}$ , the scalar $w_{i}$ represents the weight of first order feature, the latent vector $v_{i}$ denotes the weight vector of second-order feature interactions, and the related output of FM component can be calculated as follows:

$$y_{FM}=\sum_{i=1}^{n}w_{i}x_{i}+\sum_{i=1}^{n}\sum_{j=i+1}^{n}\langle v_{i},v_{j}\rangle x_{i}x_{j}$$ (7)

where 〈⋅,⋅〉 represents the inner product between two vectors, $\langle v_{i},v_{j}\rangle x_{i}x_{j}$ is the second-order interaction between the ith feature and the jth feature.

2) Deep component: Deep component is a deep neural network, which captures high-order feature interactions. For the sample x which can be expressed as an n dimension vector, the input of each layer can be formulated as follows:

$$x^{l}=\sigma \left(w^{l}x^{l-1}+b^{l}\right)\left(l=1,2,\cdots ,L\right)$$ (8)

where L denotes the number of hidden layers, σ represents an activation function, $w^{l}$ is the model weights for the lth layer, and $b^{l}$ is the model bias for the lth layer. Like the general definition of a deep neural network, the output of the Deep component can be formulated as follows:

$$y_{Deep}=w^{l+1}x^{l}+b^{l+1}$$ (9)

Note that the output layer of Deep component does not use any activation function, because ML-DeepFM is a multi-label classifier. Theoretically, we can get the predicted labels by combing Eqss 6, 7, 9, but it can be found that there are many unknown parameters in these formulas, such as v , thus we design a loss function to optimize these parameters in the process of training the FM component and Deep component. For the task of multi-label classification, we hope that the predicted scores of the relevant labels are higher than those of irrelevant labels for each sample, and the mathematical formula is as follows:

$$L_{ml}=\sum_{i=1}^{N}\sum_{k\in \Omega \left(irre\right),r\in \Omega \left(re\right)}\left(\bar{y_{i}^{k}}-\bar{y_{i}^{r}}\right)$$ (10)

where N is the number of all samples, Ω(irre) is the set of irrelevant labels, Ω(re) is the set of relevant labels, $\bar{y_{i}^{k}}$ represents the predicted scores of irrelevant labels, conversely, $\bar{y_{i}^{r}}$ denotes the predicted scores of relevant labels.

The loss function as shown in Eq. 10 has a problem that the learning rate of the FM component and the Deep component is very slow when using gradient descent for training the model. Log function is adopted to solve this problem inspired by cross entropy. In addition, it can be found that the value of $\bar{y_{i}^{k}}-\bar{y_{i}^{r}}$ is less than *0*, which means $\bar{y_{i}^{k}}-\bar{y_{i}^{r}}$ cannot be directly entered into the log function. We perform a nonlinear mapping from $\bar{y_{i}^{k}}-\bar{y_{i}^{r}}$ to $e^{\bar{y_{i}^{k}}-\bar{y_{i}^{r}}}$ , which guarantees that the input value is greater than *0*. Thus, the loss function can be expressed as follows:

$$L_{ml}=\sum_{i=1}^{N}\sum_{k\in \Omega \left(irre\right),r\in \Omega \left(re\right)}e^{\bar{y_{i}^{k}}-\bar{y_{i}^{r}}}$$ (11)

For a multi-label classifier, the number of labels for each sample is not fixed, we need a threshold value to determine the number of relevant tags to output. Assuming that the threshold value is denoted as $y^{0}$ , the loss function can be improved to:

$$L_{ml}=\sum_{i=1}^{N}\log\left(\sum_{k\in \Omega \left(irre\right),r\in \Omega \left(re\right)}e^{\bar{y_{i}^{k}}-\bar{y_{i}^{r}}}+\sum_{k\in \Omega \left(irre\right)}e^{\bar{y_{i}^{k}}-y^{0}}+\sum_{r\in \Omega \left(re\right)}e^{y^{0}-\bar{y_{i}^{r}}}\right)$$ (12)

## 3 Results and Discussion

### 3.1 Experimental Setup

In this work, the proposed Gm-PLoc is implemented with TensorFlow under the Windows 10 operating system, and all the experiments are conducted on a computing server with an Intel(R) Core(TM) i7-10700K CPU and an Nvidia GeForce RTX 3080 Ti GPU.

Gm-PLoc is composed of SM-GAN and ML-DeepFM. In SM-GAN, the generator is consistent of one dense layer and three deconvolution layers, and the discriminator includes one dense layer and two convolution layers. In ML-DeepFM, FM component includes one FM layer and one dense layer, and Deep component includes five dense layers which have 256, 128, 64, 32 and 14 neurons, respectively. During training, the networks-based models are trained with Adam optimizer for 1,000 epochs, the batch size and learning rate are set to 512 and 0.0001, respectively. Note that the reason for using deep neural networks is to automatically extract the lower-order and high-order feature interactions, at the same time, we perform the following operations to alleviate the over fitting problem caused by using deep neural networks: expanding the dataset by SM-GAN, partitioning the dataset by the 10 cross validation, building shallow networks and reducing the number of neurons by the dropout technique.

### 3.2 Performance Measure

For evaluating the performance of the proposed model Gm-PLoc, 10 fold cross validation test is selected as the assessment method, which randomly divides the dataset into ten subsets, nine of them are used for training and the rest is retained as the test data, repeating the process ten times. In the meantime, hamming loss (Hl), one-error (Oe), coverage (Co), ranking loss (Rl), and average precision (Ap) are used to quantify the performance of the proposed model for multi-label protein subcellular localization. These five performance evaluation metrics are widely used in the literature on multi-label learning (Yu and Zhang, 2021; Zhang et al., 2021), which can be defined as follows:

1) Hl evaluates the fraction of labels misclassified in the wrong classes completely. There are two situations when the labels are misclassified: relevant labels are missed and irrelevant labels are predicted, and the mathematical expression is shown in Eq. 13.

$$Hl\left(\hat{y},y\right)=\frac{1}{N}\sum_{i=1}^{N}\sum_{j=1}^{K}\left(\hat{y_{ij}}\neq y_{ij}\right)$$ (13)

2) Oe evaluates the fraction that the top-ranked label of samples is excluded from the relevant labels, which can be formulated as follows:

$$Oe\left(\hat{f},y\right)=\frac{1}{N}\sum_{i=1}^{N}\left(\left(\arg\underset{j\in \left\{1,2,\cdots ,K\right\}}{\max}\hat{f_{ij}}\right)\notin y_{i}\right)$$ (14)

3) Co evaluates the average number of steps that must be moved when the ranked label list cover the relevant labels, and related mathematical definition will be:

$$Co\left(\hat{f},y\right)=\frac{1}{N}\sum_{i=1}^{N}\underset{j:y_{ij}=1}{\max}rank\left(\hat{f_{ij}},y_{ij}\right)$$ (15)

4) Rl measures the times that the relevant labels are ranked higher than the relevant labels, which can be defined by Eq. 16.

$$Rl\left(\hat{f},y\right)=\frac{1}{N}\sum_{i=1}^{N}\frac{1}{{\left\|y_{ik}\right\|}_{0}\left(K-{\left\|y_{ik}\right\|}_{0}\right)}{\left\|\left\{\left(k,l\right):\hat{f_{ik}}\leq \hat{f_{il}},y_{ik=1},y_{il}=0\right\}\right\|}_{0}$$ (16)

5) Ap evaluates the average times that the relevant labels are ranked higher than each particular label of samples.

$$Ap\left(\hat{f},y\right)=\frac{1}{N}\sum_{i=1}^{N}\frac{1}{{\left\|y_{i}\right\|}_{0}}\sum_{j:y_{ij}=1}\frac{{\left\|L_{ij}\right\|}_{0}}{{\left\|rank\left(\hat{f_{ij}},y_{ij}\right)\right\|}_{0}}$$ (17)

where $L_{ij}=\left\{k:f_{ik}\geq f_{ij},y_{ik}=1\right\}$ $L_{ij}$ . In the above definitions, K denotes the length of real labels, N means the number of all samples in each dataset, y denotes the real labels of sample, $\hat{y}$ and $\hat{f}$ represent the label and score returned by the multi-label classifier, respectively. For a good prediction model, the value of Hl, Oe, Co and Rl should be as small as possible, but the value of Ap should be the opposite.

### 3.3 The Analysis of Imbalance Degree on the Dataset

**FIGURE 4:**
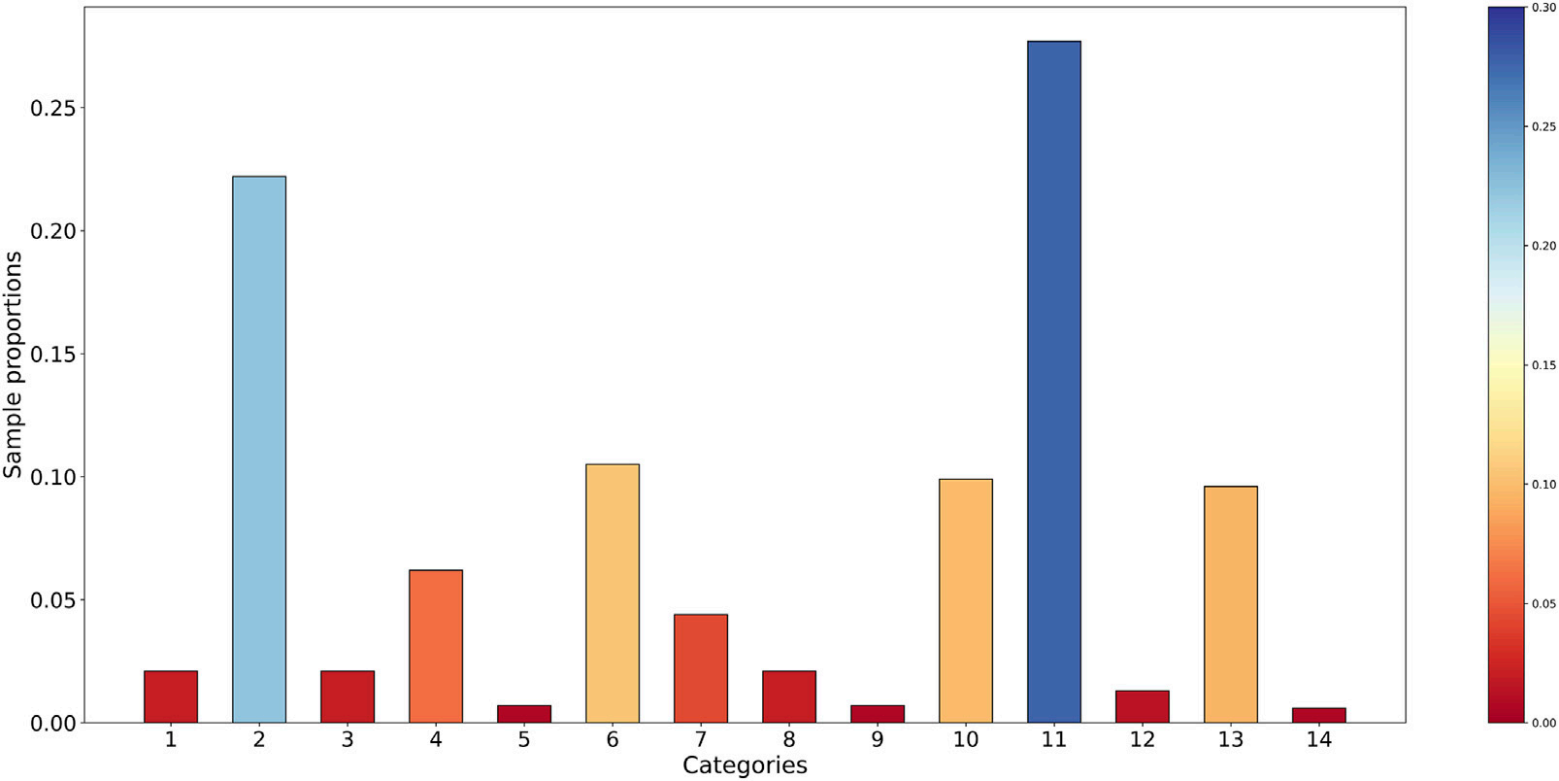
The sample proportion of the benchmark dataset.

There is a strong connection between the imbalance degree of the protein dataset and the performance of the classifier. The imbalance degree of the dataset is performed through numerical representation in Figure 4. The abscissa corresponds to the categories of subcellular localization, and the ordinate represents the proportion of each category. The bigger the value of sample proportion is, the more the number of samples in the relevant category is, or the other way around.

As shown in Figure 4, the phenomenon of class imbalance exists in the benchmark dataset. The sample proportions of eight categories are all lower than 0.05, while two categories are higher than 0.20. It can be found that there is a large gap in the number of samples between different categories, and the data distribution among multiple categories has the imbalance problem. In combination with the imbalance ratio, this benchmark dataset is considered extremely imbalanced.

### 3.4 The Analysis of the Performance of SM-GAN and ML-DeepFM

To assess the performance of SM-GAN and ML-DeepFM proposed in this paper, we conduct six controlled experiments obtained by combining SM-GAN and ML-DeepFM in pairs. Note that here ML-DeepFM is divided into two sub-classifiers, ML-Deep and ML-FM, to explore the relationship between the Deep component, FM component and ML-Deep. Moreover, the results of each controlled experiment are fed back through five metrics.

**FIGURE 5:**
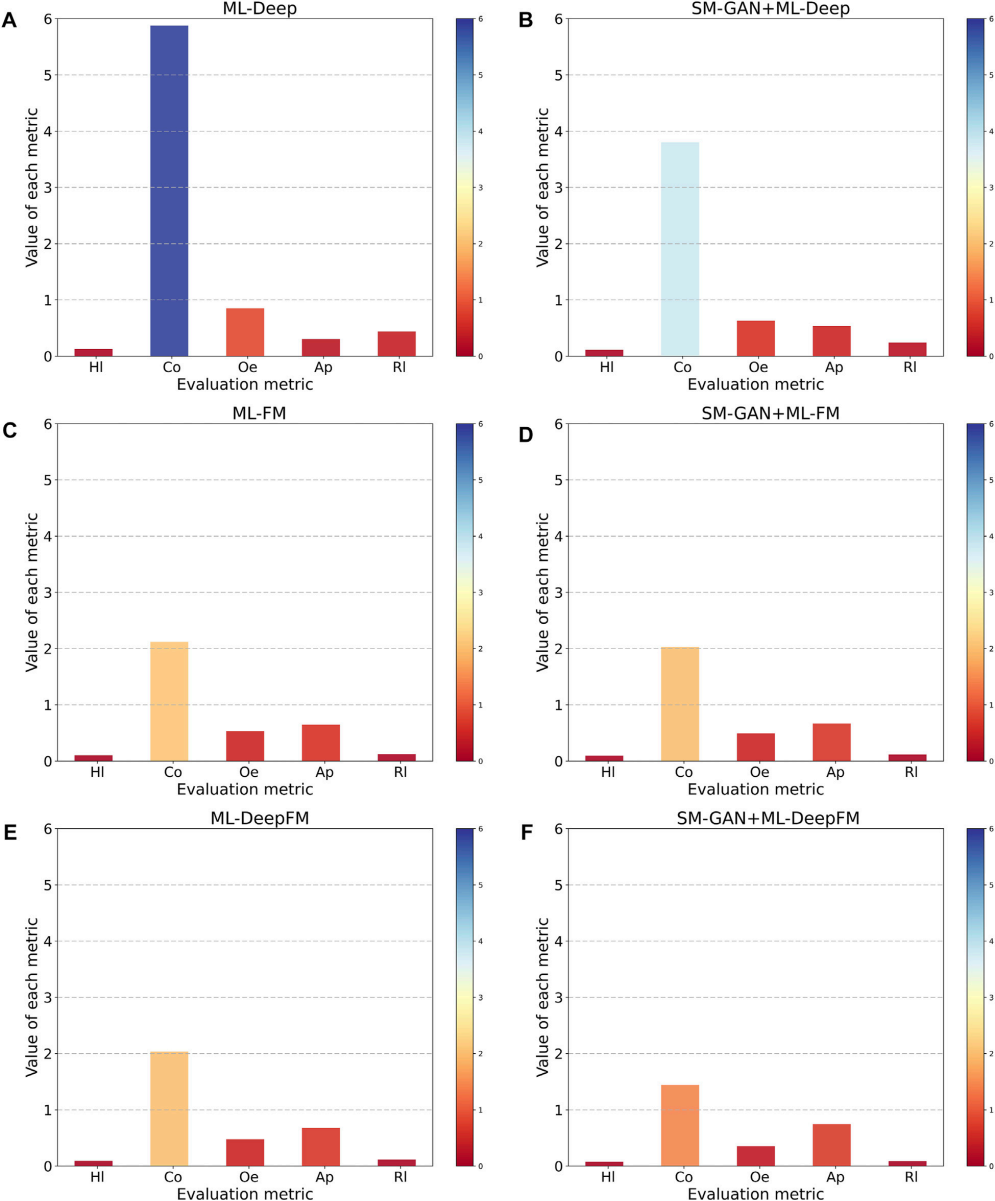
The relationship between SM-GAN with ML-DeepFM.

**FIGURE 6:**
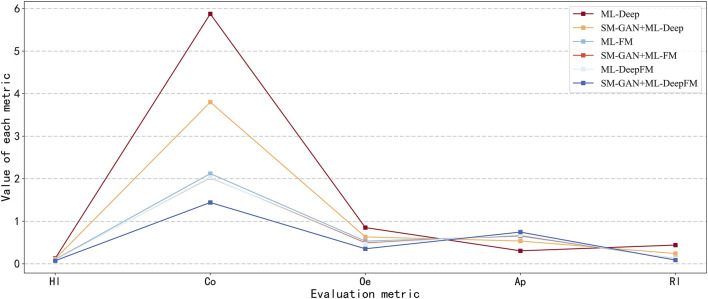
The experimental results come from the exhaustive combination of SM-GAN and ML-DeepFM.

From two dimensions of SM-GAN and ML-DeepFM, this paper analyzes the results of each controlled experiment, and the relevant results are shown in Figure 5 and Figure 6. Where Figure 5 includes six subgraphs. In Figure 5, the (b), (d) and (f) in the right column represent the results obtained after rebalancing by SM-GAN, we can see that the value of Co in the subgraph (b) is significantly smaller than that in the subgraph (a), and the above situation also exists in subgraph (e) and (f), which means SM-GAN can effectively promote the classifier to completely recognize the relevant labels of each sample. In addition, the rest metrics after rebalancing are better than other controlled experiments in varying degrees. The experimental results further prove the negative impact of data imbalance for the classifier, and also verify the effectiveness of SM-GAN.

To evaluate the performance of ML-DeepFM, and analyze the contribution of Deep component and FM component in ML-DeepFM, the experimental results of six controlled experiments are summarized in Figure 6. The ML-Deep gets the worst results as shown in the red line in that the values of Hl, Co, Oe and Rl are the largest and the value of Ap is the lowest. On the contrary, the results obtained by SM-GAN + ML-DeepFM are the best. In addition, we can see that the performance of classifiers based on ML-FM is better than those based on ML-Deep, and is close to that of ML-DeepFM, which means the FM component contributes more to ML-DeepFM and the information of low-order feature interactions between protein sequences is more useful for predicting subcellular localization.

### 3.5 Performance Comparisons Between Different Oversampling Methods

As an oversampling method for generating samples of the minority classes, SM-GAN can alleviate the class imbalance problem in protein subcellular localization. To assess the effectiveness, we compare SM-GAN against other popular methods that can alleviate class imbalance problem. Among these controlled methods, SMOTE (Buda et al., 2018) generates new samples of the minority class through linear interpolation, and Borderline-SMOTE (Han et al., 2005) is a variant of SMOTE, which only samples the boundary samples of the minority class. SVM-Balance (Farquad and Bose, 2012) uses SVM to solve the problem of data imbalance by reconstructing the training dataset. SMOTE, Borderline-SMOTE, and SVM-Balance are conventional methods for imbalanced learning. The remaining methods including SinGAN (Shaham et al., 2019) and DCGAN (Yu Y. et al., 2017) are both generative models based on GAN, which generate samples by learning the sample distribution of the minority class. The experimental results of the above mentioned methods can be found in Table 1.

**TABLE 1:** Comparing SM-GAN with other oversampling methods.

Method for imbalanced learning	Hl	Co	Oe	Ap	Rl
SMOTE	0.106	2.050	0.552	0.626	0.121
Borderline-SMOTE	0.102	2.138	0.521	0.646	0.126
SVM-Balance	0.105	1.998	0.546	0.635	0.117
SinGAN	0.089	1.983	0.463	0.684	0.116
DCGAN	0.083	1.669	0.452	0.696	0.102
SM-GAN	**0.073**	**1.441**	**0.353**	**0.745**	**0.087**

As shown in Table 1, experimental results corresponding to the methods based on the generative model are much better than conventional methods, because these interpolation methods based on sample spacing will further enhance the stacking of samples, thereby making correct classification difficult. In addition, among the three imbalanced learning methods based on the generative model, the values of five evaluation metrics corresponding to SM-GAN are the best. To sum up, SM-GAN can effectively handle the extreme imbalance problem of multi-label samples, which performs better than compared methods.

### 3.6 Performance Comparison With Existing Prediction Models for Protein Subcellular Localization

To further verify the effectiveness of Gm-PLoc obtained by combining SM-GAN and ML-DeepFM, we compare the performance of Gm-PLoc with GO + AAC + PseAAC + IMMMLGP (He et al., 2012), GO + FunD + PSSM + OET-KNN (Shen and Chou, 2009) and PSSM + PseAAC + Multi-SVM (Shen et al., 2019) on the same benchmark dataset. For the sake of simplicity, “+” is used to connect the feature representation and classifier in the corresponding models. The experimental results of Gm-PLoc compared with the three methods are shown in Table 2.

**TABLE 2:** Comparing Gm-PLoc with other model of protein subcellular localization.

Model of subcellular localization	Co	Ap	Rl
GO + AAC + PseAAC + IMMMLGP	4.303	0.581	0.419
GO + FunD + PSSM + OET-KNN	5.317	0.579	0.496
PSSM + PseAAC + Multi-SVM	1.719	0.706	0.108
PSSM + SM-GAN + ML-DeepFM	**1.441**	**0.745**	**0.087**

The bold values provided in Table 2 mean the best results calculated with different protein subcellular localization methods.

From Table 2, we can see that the values of Co and Rl calculated by Gm-PLoc are 1.441 and 0.087, respectively, which are 0.278–2.862 and 0.021–0.409 lower than those of other models. In addition, the value of Ap is 0.745, which is 0.039–0.166 higher than the comparison models. And by comparing all metrics, it can be found that the Gm-PLoc has the best performance. The above results further elucidate that Gm-PLoc performs better than the comparison models.

Furthermore, we analyze the comparison models and find that most of these models improve the accuracy of subcellular localization by fusing features and improving algorithms. These models do not consider the problem of data imbalance, while Gm-PLoc pays attention to the feature information, classification model and imbalanced characteristic simultaneously. Overall, Gm-PLoc can predict subcellular localization effectively.

## 4 Conclusion

The class imbalance problem widely exists in the datasets annotated with the subcellular localization, which seriously affects the performance of classification models for multi-label proteins. In this paper, a new model named Gm-PLoc is proposed to solve the problem of the multi-label classification with imbalanced protein data. In this model, the evolution information of proteins is extracted by the PSSM, and then the proposed SM-GAN is used to rebalance the distribution of each class. Finally, ML-DeepFM based on DeepFM is trained with the rebalanced dataset to predict the subcellular localization of multi-label proteins. Many experiments are conducted to assess the performance of Gm-PLoc, and the experimental results illustrate that Gm-PLoc can alleviate the problem of protein data imbalance and predict the subcellular localization of multi-label proteins effectively. In the field of protein subcellular localization, there are two aspects may be improved further: the processing of protein data and the construction of multi-label classification. In further works, we will explore the more effective and cost efficient classification model for protein subcellular localization under the data imbalance and big categories.

## Data Availability

Publicly available datasets were analyzed in this study. This data can be found here: https://github.com/WULIWEN-007/GmPLoc-Frontiers.
